# Dose escalation to high-risk sub-volumes based on non-invasive imaging of hypoxia and glycolytic activity in canine solid tumors: a feasibility study

**DOI:** 10.1186/1748-717X-8-262

**Published:** 2013-11-07

**Authors:** Malene M Clausen, Anders E Hansen, Per Munck af Rosenschold, Andreas Kjær, Annemarie T Kristensen, Fintan J McEvoy, Svend A Engelholm

**Affiliations:** 1 Department of Radiation Oncology, Rigshospitalet, Section 3994, Blegdamsvej 9, 2100 Copenhagen, Denmark; 2 Department of Clinical Physiology, Nuclear Medicine & PET and Cluster for Molecular Imaging, Blegdamsvej 9, 2100 Copenhagen, Denmark; 3 Department of Veterinary Clinical and Animal Sciences, Dyrlægevej 16, 1870 Frederiksberg C, Denmark; 4 Technical University of Denmark, DTU Nanotech, Center of Nanomedicine and Theranostics, Produktionstorvet, building 423, 2800 Kgs. Lyngby, Denmark; 5 Niels Bohr Institute, Blegdamsvej 17, 2100 Copenhagen, Denmark

## Abstract

**Introduction:**

Glycolytic activity and hypoxia are associated with poor prognosis and radiation resistance. Including both the tumor uptake of 2-deoxy-2-[^18^ F]-fluorodeoxyglucose (FDG) and the proposed hypoxia tracer copper(II)diacetyl-bis(N^4^)-methylsemithio-carbazone (Cu-ATSM) in targeted therapy planning may therefore lead to improved tumor control. In this study we analyzed the overlap between sub-volumes of FDG and hypoxia assessed by the uptake of ^64^Cu-ATSM in canine solid tumors, and evaluated the possibilities for dose redistribution within the gross tumor volume (GTV).

**Materials and methods:**

Positron emission tomography/computed tomography (PET/CT) scans of five spontaneous canine solid tumors were included. FDG-PET/CT was obtained at day 1, ^64^Cu-ATSM at day 2 and 3 (3 and 24 h pi.). GTV was delineated and CT images were co-registered. Sub-volumes for 3 h and 24 h ^64^Cu-ATSM (Cu3 and Cu24) were defined by a threshold based method. FDG sub-volumes were delineated at 40% (FDG40) and 50% (FDG50) of SUV_max_. The size of sub-volumes, intersection and biological target volume (BTV) were measured in a treatment planning software. By varying the average dose prescription to the tumor from 66 to 85 Gy, the possible dose boost (*D*_*B*_) was calculated for the three scenarios that the optimal target for the boost was one, the union or the intersection of the FDG and ^64^Cu-ATSM sub-volumes.

**Results:**

The potential boost volumes represented a fairly large fraction of the total GTV: Cu3 49.8% (26.8-72.5%), Cu24 28.1% (2.4-54.3%), FDG40 45.2% (10.1-75.2%), and FDG50 32.5% (2.6-68.1%). A BTV including the union (∪) of Cu3 and FDG would involve boosting to a larger fraction of the GTV, in the case of Cu3∪FDG40 63.5% (51.8-83.8) and Cu3∪FDG50 48.1% (43.7-80.8). The union allowed only a very limited *D*_*B*_ whereas the intersection allowed a substantial dose escalation.

**Conclusions:**

FDG and ^64^Cu-ATSM sub-volumes were only partly overlapping, suggesting that the tracers offer complementing information on tumor physiology. Targeting the combined PET positive volume (BTV) for dose escalation within the GTV results in a limited *D*_*B*_. This suggests a more refined dose redistribution based on a weighted combination of the PET tracers in order to obtain an improved tumor control.

## Introduction

Intensity-modulated radiotherapy (IMRT) has allowed for more complex dose distributions. One of the challenges in radiation oncology research is to determine the therapeutic potential of advanced imaging combined with non-uniform and very accurate dose delivery [1].

In conventional radiotherapy (RT) the dose is delivered homogeneously to the target volume. However, solid tumors are often very heterogeneous with a regionally variable radiosensitivity, and the tumor control might therefore be increased with a non-uniform dose distribution [2]. It is hypothesized that a redistribution of the dose can potentially increase the tumor control probability (TCP) without increasing radiation toxicity [2-4].

Positron emission tomography (PET) can provide spatial information on tumor characteristics associated with radioresistance, and including this modality in RT planning would allow a more refined characterization of individual tumors and thereby a tailored prescription of radiation dose [2]. In 2000, Ling et al. proposed the concept of combining biological images (e.g. PET with multiple tracers) and thereby targeting the union of different sub-volumes, in a “biological target volume” (BTV) [5].

Bentzen has introduced the concept of theragnostic imaging, referring to the use of information from functional and molecular imaging to determine individual treatment of patients [1]. The successful clinical introduction of this concept depends on the ability of the diagnostic method to visualize tumor sub-volumes that are potentially radioresistant, e.g. hypoxic areas, and the ability of delivery methods to apply a higher dose to that specific volume. The aim of such “dose painting” is reaching biological conformity of the dose delivery to tumor. In dose painting by contours (DPBC) a higher, homogenous dose is prescribed to the functional part of the gross tumor volume (GTV) delineated on computed tomography (CT) and thereby distinguishing high-risk from low-risk areas [6]. This strategy is clinically feasible as commercial treatment planning software can be used and uncertainties can be accounted for by addition of margins [7]. Dose painting by numbers (DPBN) is a more sophisticated strategy where the dose prescription is based on information from every voxel [1,8]. Both dose painting strategies can be used to substantially increase the dose to specific regions in the tumor. However, by using the DPBN approach, a higher peak dose can be obtained, which might have a positive impact on TCP [7].

A large boost volume may limit the option for escalation to a high peak dose due to normal tissue constraints. Targeting a BTV, defined by the DPBC strategy, will inevitably lead to a larger boost volume than targeting a sub-volume based on one PET tracer, and potentially thereby limit the possibility for dose redistribution. The inclusion of more radioresistant regions for RT, can therefore possibly lead to a decreased tumor control.

Glycolytic activity and hypoxia are examples of biologic targets for RT treatment since they are both associated with a malignant phenotype and poor prognosis [9-12]. Hypoxia upregulates numerous glycolytic genes and 2-deoxy-2-[^18^ F]-fluorodeoxyglucose (FDG) has therefore been suggested has as a marker of tumor hypoxia [13]. However, several promising hypoxia specific endogenous markers and PET tracers have been introduced, and it can be hypothesized that including the uptake of both FDG and a hypoxia tracer in targeted therapy may lead to an improved TCP. The correlation between FDG uptake and hypoxia markers has been extensively studied, but the results have been inconsistent and generally failed to consistently link a high uptake of FDG with tumor hypoxia [14-18]. The most recent studies have evaluated the spatial correlation between FDG and hypoxia tracers by using isolated tumor cell lines, two-dimensional pixel-by-pixel comparisons of either autoradiography or PET based images, some with microscopic validation [13-16,19,20].

The overall conclusion seems to be that FDG is not a good marker of hypoxia, but that both FDG and hypoxia tracers can provide useful, possibly complementary, information regarding prognosis. These studies provide valuable information on tracer uptake and tumor microenvironment. However, a more pragmatic approach on integrating this information in RT planning has not been explored. Translating a voxel-based PET signal into a threshold-based RT target volume might impact the correlation between tracers. Therefore, the question is whether the observed mismatches between FDG uptake and hypoxia in the studies mentioned above also make FDG a poor marker of hypoxia in threshold-based RT planning.

In this study we analyzed the correlation between the PET positive sub-volumes of FDG and the hypoxia tracer copper(II)diacetyl-bis(N^4^)-methylsemithiocarbazone (Cu-ATSM) at two different time-points after injection in spontaneous canine tumors.

Using a threshold based method for target definition introduces several uncertainties and might impact the results. Various cut-off values for FDG uptake have previously been used, but the ideal cut-off has not been defined. ^64^Cu-ATSM is a rather novel hypoxia tracer, and the distribution kinetics and image acquisition time is not completely clarified. In order to investigate these aspects, we included different cut-off values for FDG and two different time-points for ^64^Cu-ATSM imaging in this analysis.

The aim of this study was to evaluate FDG as a marker of hypoxia in a DPBC approach, and to assess the feasibility for redistribution of dose by a DPBC strategy that includes the PET tracers FDG and ^64^Cu-ATSM.

## Methods and materials

### Tumor imaging and image processing

This study was based on PET/CT scans of five spontaneous canine sarcomas and carcinomas. Subjects were scanned prior to and as part of the planning of subsequent radiation therapy, and according to a prospective protocol approved by the local ethics committee [21]. PET/CT images were obtained using a combined PET/CT scanner (Biograph 40, Siemens, Munich, Germany), consisting of a high-resolution PET-scanner and a 40-slice CT-scanner. The CT scanning parameters were: slice thickness of 3.0 mm, 120 kV, 170 mAs, pitch 1.2, collimation 24 × 1.2 mm and a B30 kernel. The PET images were acquired using a 3D acquisition mode and a 3D OSEM reconstruction algorithm with sections of 8 subsets and 4 iterations and smoothed using a Gaussian filter with 3-mm FWHM, and a matrix size of 256 × 256.

The dogs were scanned on three consecutive days, ^18^ F-FDG at day 1 (approx. 1 hour p.i.) and ^64^Cu-ATSM at day 2 and 3 (approx. 3 and 24 hours pi.). This scanning protocol allowed sufficient radioactive decay and tracer washout between imaging sessions with the two tracers. Dogs received a mean activity of 7.7 MBq/kg of both tracers.

### Tumor and sub-volume delineation

All scans were transferred to a radiotherapy treatment planning software, Eclipse v10 (Varian Medical Systems, Palo Alto, CA, US). The GTV was delineated on the day 1 CT in collaboration with an experienced radiologist and a veterinarian. CT images were manually co-registered in a treatment planning software image registration (Eclipse, Varian Medical Systems, Palo Alto, US) with the day 2 CT-scan as the reference image. The PET uptake was used to define glycolytic and hypoxic sub-volumes. The hypoxic sub-volume was defined based on a study performed by Bowen et al. [22], who developed transformation function curves between 3 h ^64^Cu-ATSM uptake and tissue oxygen tension. The SUV that reflected tumor hypoxia (defined as pO_2_ < 10 mmHg) was 1.4. This cut-off value was used to define the hypoxic sub-volume for the 3 h ^64^Cu-ATSM in this study (Cu3). No previous studies have investigated a cut-off value for the uptake of ^64^Cu-ATSM 24 h p.i., and the 24 h ^64^Cu-ATSM sub-volume (Cu24) was therefore defined based on calculation of the corresponding tumor-to-muscle ratio (T/M), which was assumed constant.

Based on previous studies, two FDG sub-volumes were delineated at 40% (FDG40) and 50% (FDG50) of SUV_max_[23,24]. Boolean operators function was used to measure all sub-volumes including the intersection of the sub-volumes and the total BTV defined as the union of ^64^Cu-ATSM and either FDG40 or FDG50.

### Dose redistribution

One strategy for dose redistribution is to use pre-treatment PET data to increase the dose in potentially radioresistant areas. We explored this strategy assuming that: 1) the volumes are best selected using a DPBC approach by a cut-off value 2) the integral dose to the GTV is kept constant which would tend to keep toxicity at a proximity constant level, and 3) a “base dose” of 66 Gy to the CT-defined tumor needs to be maintained in order to obtain tumor control. In reality, the dose distribution within the GTV will also affect the surrounding normal tissue [25]. Situations may occur where a high dose region is present in the periphery of the GTV, and this can be problematic in cases where a serial responding organ is closely related to the GTV. In a clinical setting dose constraints would be applied for organs at risk.

By varying the average dose prescription (*P*) to the tumor from 66 Gy to 85 Gy, the possible dose increase (“boost dose”, *D*_*B*_) upholding the assumptions 1–3 above for one or multiple PET tracers can be calculated. An average GTV dose above 85 Gy is probably unwarranted in most clinical scenarios and was therefore used as the upper limit in this work. The boost increase is derived assuming that the optimal target for boost dose equals one of, the union of or the intersection of the PET tracers, using the volumetric data for the PET tracers (*V*_*i*_) and the total GTV.

It follows from assumptions 1–3 that

$$P=D_{B}\;x\;\left(\mathrm{Vi}/\mathrm{GTV}\right)+66\;x\;\left(1-\mathrm{Vi}/\mathrm{GTV}\right)$$

The ratio (R) of dose increase (“boost ratio”) is thus

$$R=D_{B}/P=\left(P–66\;x\;\left(1-\mathrm{Vi}/\mathrm{GTV}\right)\right)/\left(P\;x\;\mathrm{Vi}/\mathrm{GTV}\right)$$

## Results

**Figure 1:**
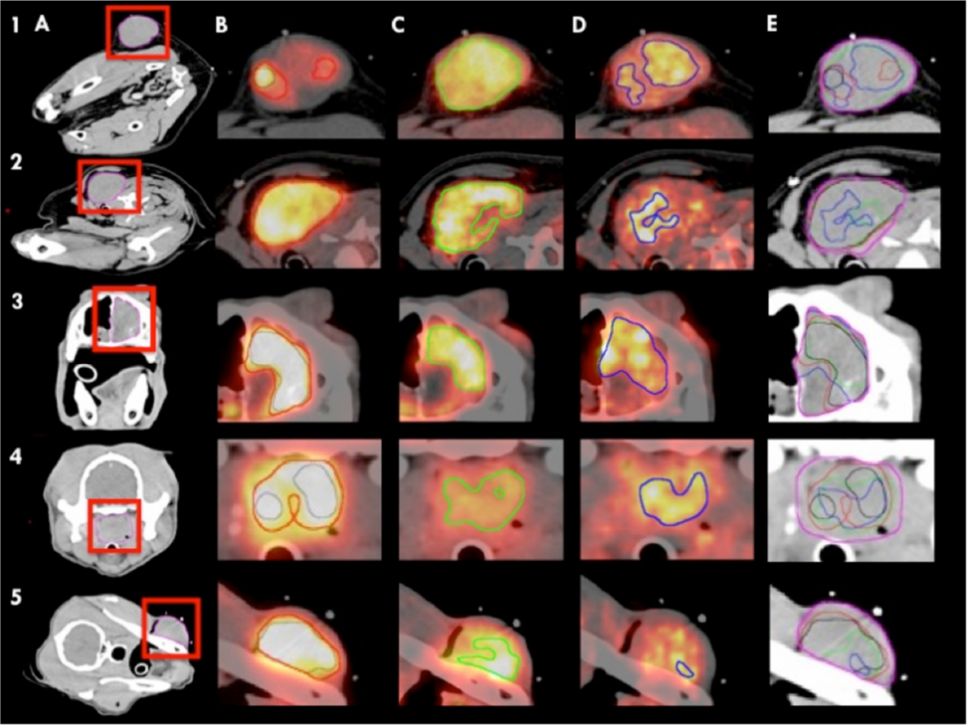
**Tracer uptake, distribution and sub-volume definition.** The dogs are horizontally listed 1–5. The images in column **A** show the tumor location and GTV (pink). **B**: FDG40 (red) and FDG50 (black), **C**: Cu3 (green), **D**: Cu24 (blue). Column **E** shows all sub-volumes and their respective overlap. The tendency of the Cu24 sub-volume to be included in either the FDG sub-volume or Cu3 is illustrated here.

The SUV_max_ measurements for FDG PET and ^64^Cu-ATSM PET ranged from 7.9-23.1 (FDG) and 2.3-3.6 (Cu3). The cut-off value of SUV 1.4 defining the Cu3 sub-volume in this study corresponded to 50% of SUV_max_. PET/CT images and the sub-volumes delineated are depicted in Figure 1 for all five cases. All defined sub-volumes were measured by volume and their fraction of the total GTV was calculated. The overlap or intersection between FDG and ^64^Cu-ATSM was described by the intersection volume (∩), which is the volume included in both tracer sub-volumes. The union (∪) of both tracers representing the BTV was also measured. The patient characteristics and sub-volume data are outlined in Table 1.

**Table 1:** Patient characteristics and sub-volume data

**Dog no.**	**Tumor type**	**Tumor localization**	**GTV (ccm)**	**FDG40 (ccm)**	**FDG50 (ccm)**	**Cu3 (ccm)**	**Cu24 (ccm)**	**Cu3∪FDG40 (ccm)**	**Cu3∪FDG50(ccm)**	**Cu3∩FDG40/FDG40 (%)**	**Cu3∩FDG40/Cu3 (%)**	**Cu3∩FDG50/FDG50 (%)**	**Cu3∩FDG50/Cu3 (%)**
1	Hemangiopericytoma	Lumbar region	128.7	13.0	3.4	85.8	61.6	86.1	85.9	98.5	14.9	98.2	3.9
2	Fibrosarcoma	Lat. cervical region	88.2	66.4	60.1	63.9	17.0	73.9	71.3	84.7	88.4	88.2	82.8
3	Squamous cell carcinoma	Nasal cavity	55.4	23.7	17.0	21.2	30.0	28.7	25.4	68.9	76.9	76.4	61.0
4	Adenocarcinoma	Nasopharynx	20.8	10.3	5.3	9.3	3.5	13.0	10.7	62.9	69.3	75.4	42.8
5	Undiff. soft tissue sarcoma	Mandible	23.2	11.2	8.4	6.2	0.6	12.2	10.2	46.6	83.9	52.8	70.8
Avg.			63.3	24.9	18.8	37.3	22.5	42.8	40.7	72.3	66.7	78.2	52.3

**Figure 2:**
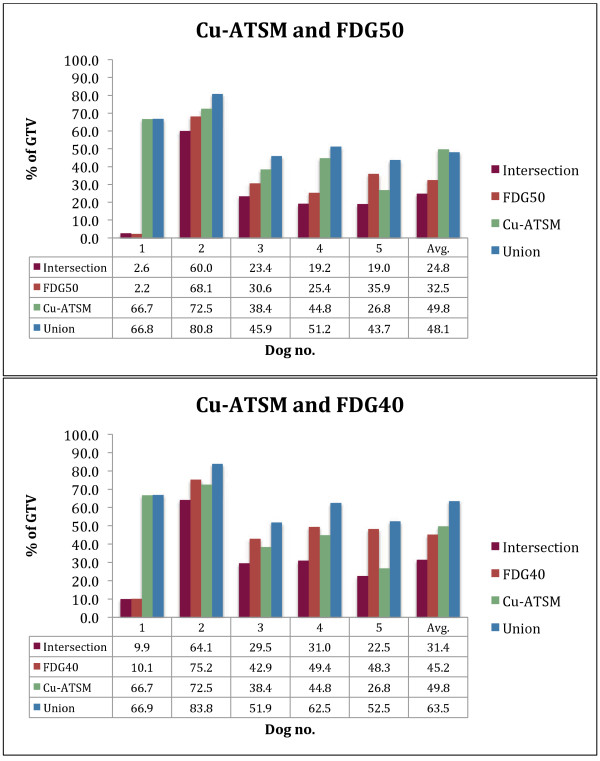
**Sub-volume fraction of the total GTV for **^**64**^**Cu-ATSM (Cu3), FDG40 and FDG50.**

**Figure 3:**
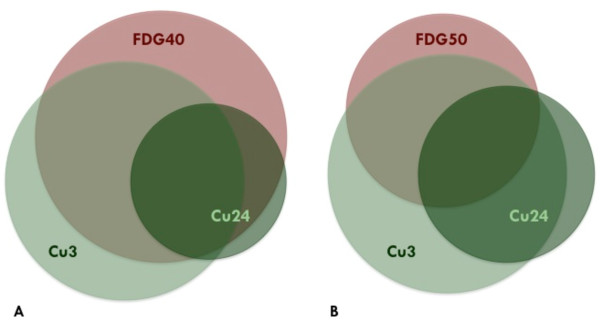
**The proportional PET positive volume of FDG40, Cu3 and Cu24, and their respective overlap is shown in A. B** shows the overlap between FDG50 and the two ^64^Cu-ATSM sub-volumes. The areas and the overlap drawn schematically correspond to the average volumetric size (% of GTV) found in the study.

The potential boost volumes represent a fairly large fraction of the total GTV: Cu3 49.8% (26.8-72.5%), Cu24 28.1% (2.4-54.3%), FDG40 45.2% (10.1-75.2%), and FDG50 32.5% (2.6-68.1%). Figure 2 shows the fraction of GTV for all sub-volumes and illustrates the large variation between the cases. The mean values and proportional PET positive volume of FDG40, Cu3 and Cu24, and their respective overlap is shown in Figure 3A. Figure 3B shows the overlap between FDG50 and the two Cu-ATSM sub-volumes.

The data reflects a general trend that a large proportion of Cu24 is included in either Cu3 or FDG. Cu24 is included in the Cu3∪FDG40 BTV in three out of five cases, whereas the Cu3∪FDG50 BTV encompasses Cu24 in two cases. On average, 3.5% of the Cu24 sub-volume is not included in the Cu3∪FDG40 BTV, and the Cu3∪FDG50 BTV does not encompass 5.2% of Cu24. Only in one patient the Cu24 sub-volume is larger than that of Cu3.

In the following, only the relationship between Cu3 and the two FDG sub-volumes, FDG40 and FDG50, is analyzed.

Cu3∩FDG40 covered 72.3% (46.6-98.5%) of the FDG40 volume and 66.7% (14.9-88.4%) of the Cu3 volume. The Cu3∩FDG50 covered 78.2% (52.8-98.2%) of FDG50 and 52.3% (3.9-82.8%) of Cu3.

**Figure 4:**
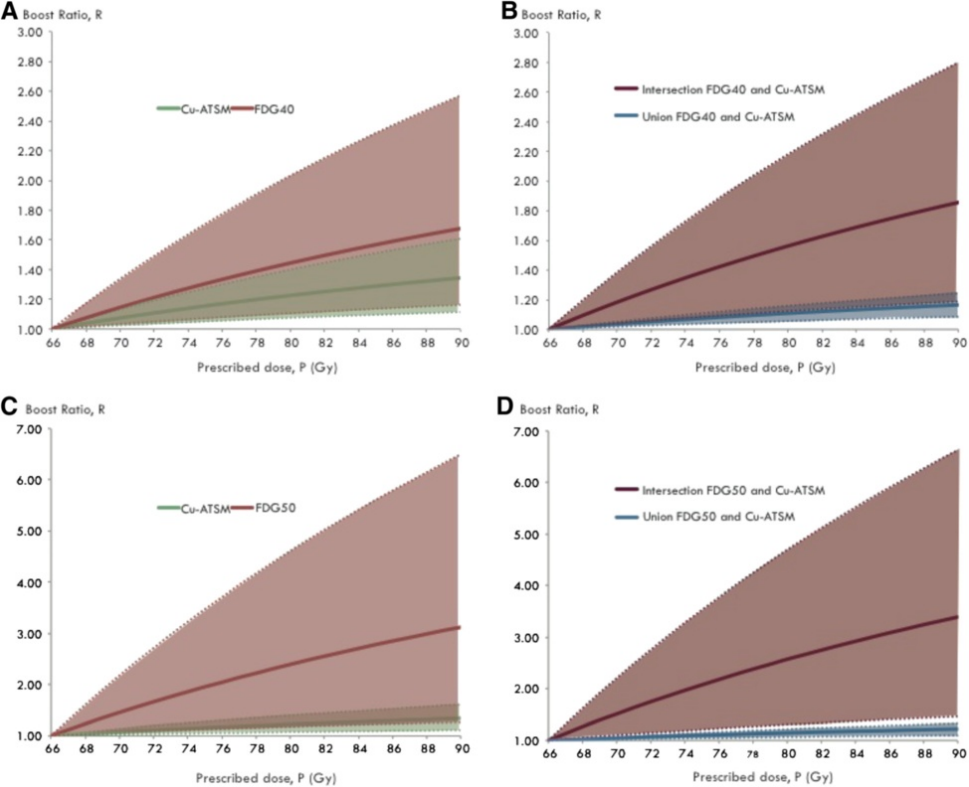
**Boost ratio as a function of dose prescription.** Dose escalation sub-volumes for ^64^Cu-ATSM, FDG40 and FDG50 are shown on the left **(A + C)**. Intersection and union volumes of the tracers are depicted on the right **(B + D)**. Cu-ATSM refers to dose escalation to the Cu3 sub-volume. Intersection and union boosts also include the two FDG sub-volumes and Cu3. The solid lines represent mean values based on all 5 dogs, and the range (80%) is illustrated in shade colors.

A BTV based on the chosen cut-off values for Cu3 and FDG involves boosting to a large fraction of the total GTV, in the case of Cu3∪FDG40 that fraction is 63.5% (51.8-83.8) and for Cu3∪FDG50 48.1% (43.7-80.8). The BTV allows only a very limited *D*_*B*_ whereas the intersection, which is a smaller volume, allows a substantial dose escalation. Figure 4 shows the possible dose escalation to sub-volumes when varying the average prescribed dose to the GTV.

## Discussion

In this study, tumor sub-volumes were defined based on a DPBC strategy of FDG and ^64^Cu-ATSM PET, and the overlap between these sub-volumes was analyzed.

Two cut-off values for FDG uptake were explored, and ^64^Cu-ATSM uptake was analyzed by sub-volumes based on two different time-points for image acquisition. We observed an overlap between all ^64^Cu-ATSM and FDG sub-volumes. However, the degree of overlap varied between cases.

Our results showed that a fairly large part of the hypoxic sub-volume as defined by ^64^Cu-ATSM was not included in a boost volume based on FDG uptake with the chosen thresholds. However, the hypoxic sub-volume did not encompass the FDG uptake either, and it therefore seems attractive to include both tracers in RT planning considering their association to treatment outcome and prognosis.

The Cu24 sub-volume was predominantly included in the BTV consisting of FDG (FDG40 or FDG50) and Cu3. This result suggests that the temporal variations of ^64^Cu-ATSM uptake might be taken into account by targeting a BTV consisting of FDG and Cu3 [21].

Several studies have linked FDG avidity or a high uptake of hypoxia tracers with poor outcome [9,26-28], and thereby confirmed the rationale for dose escalation protocols. Furthermore, a recent pattern of failure analysis in head and neck cancer patients compared FDG uptake before RT with local recurrence and revealed a higher recurrence density in FDG avid regions [29].

In theory, all tumor cells can be eradicated by a sufficiently high radiation dose. However, dose escalation must be balanced against the risk of early and late toxicities of the neighboring organs. Toxicities limit the possible escalation to large volumes, and redistribution of radiation dose might address this issue [30]. Dose escalation to potentially radioresistant areas of a tumor has proven clinically feasible in a DPBC approach without violating the constraints for the organs at risk [31,32]. The levels of dose escalation are still not clarified, and arbitrary dose prescriptions are often used in planning studies [6].

In this study, the average dose prescription could vary from 66 Gy to 85 Gy. Naturally, increasing the dose prescription level increases the room for dose redistribution from the uniform or conventional homogeneous distribution within the GTV. The boost ratio, *R*, therefore increases with increasing dose prescription, *P*. Selecting either FDG or ^64^Cu-ATSM generates more room for dose escalation, however, a part of the biological target might be missed. Conversely, a proper definition of the BTV could potentially allow for dose de-escalation in other parts of the tumor. Identification of tumor subgroups that are associated with a good prognosis also enables dose de-escalation and thereby reduced toxicity [33]. Currently, clinical trials are recruiting patients for dose de-escalation trials in human papilloma virus-positive head and neck cancer patients [34].

The fact that commercial treatment planning software can be used and margins added, makes DPBC an attractive strategy. However, this approach may lead to a steep dose gradient outside the target, and since radio responsiveness is not binary, it can be argued that the threshold based target volume definition is not biologically intuitive. Despite a relatively steep dose falloff, tumor motion require for margins to be added on both high and low dose volumes to ensure delivery of prescribed dose. Furthermore, validations of the optimal cut-off values to use are currently lacking [6,7].

To a certain extent, this study demonstrates some of the drawbacks of the DPBC approach. Using this strategy for targeting multiple biological characteristics of the tumor in a BTV will limit the possibilities for dose escalation based on the idea of a redistribution of absorbed dose. The BTV is large and adopting that volume causes the boost volume to become a significant part of the GTV, allowing for only a very limited dose redistribution and escalation to the boost region. Selecting instead the intersection, the volume becomes small allowing a substantial dose escalation and, simultaneously, the variation within the subjects becomes very large as indeed, as illustrated in Figure 4. However, most of the PET positive volume is not covered by a dose escalation to the intersection volume, and it can therefore be argued that high control rates cannot be achieved when targeting a BTV by the DPBC strategy. The more complex DPBN approach allows a linear relationship between dose prescription and functional imaging data. A recent planning study in lung cancer patients compared the two strategies, and showed that significantly higher boost doses could be achieved with DPBN [7]. The heterogeneity of hypoxia is also a motivation for targeting multiple small sub-volumes or using the DPBN strategy. However, the effect of dose falloff might be a limitation for dose escalation in small tumors, where IMRT cannot produce a dose gradient steep enough to respect adjacent normal tissue. In some cancers, brachytherapy therefore might be superior to dose escalation with IMRT [35].

Under the given assumptions for dose prescription and dose redistribution in this study, the boost volumes appear to be either too small or too large with respect to the total GTV. A more effective approach for dose redistribution from the uniform or conventional dose prescription scheme might be a DPBN strategy, where the dose prescription can be allowed to smoothly follow one or a weighted combination of PET tracers.

There are several limitations to this study, including a low number of cases, varying localizations and histopathological origin of the tumors. The DPBC approach used for analysis of sub-volume overlap is based on thresholding of PET images, which might influence the findings. We used an absolute SUV of 1.4 as cut-off value based on data from a previous study [22], and in our study this value corresponded to 50% of SUV_max_, which is a commonly used threshold for FDG. However, ^64^Cu-ATSM is a novel PET tracer and future research still needs to establish the most clinically relevant cut-off value that differentiates hypoxic from non-hypoxic areas. The possible dose increase to sub-volumes display great variation between the cases, and further studies on a more homogeneous group of tumors are therefore needed in order to elucidate the possibilities for dose targeting a BTV in RT planning. Thorough investigation of ^64^Cu-ATSM regarding distribution and uptake characteristics is also required.

## Conclusion

Based on this study of canine solid tumors FDG is not a marker of hypoxia when using the DPBC strategy. Including a BTV consisting of both FDG and the hypoxia tracer ^64^Cu-ATSM in RT planning seems attractive, as both tracers are associated with a poor prognosis. Sub-volumes representing a large fraction of the GTV only allow a limited dose redistribution and escalation, and targeting the BTV might require further dose redistribution based on a weighted combination of the PET tracers in order to obtain the optimal tumor control.

## Competing interests

The authors declare that they have no competing interests.

## Authors’ contributions

MMC participated in the design of the study, did the image and data analysis and drafted the manuscript. AEH participated in the design of the study and performed the PET/CT scans. PMR participated in the design of the study and assisted in the dose escalation calculations, and helped drafting the manuscript. AK conceived the study, and participated in its design and coordination and helped to draft the manuscript. ATK conceived the study and participated in its design. FJM helped with the image analysis and segmentation of images. SAE conceived the study, and participated in its design and coordination. All authors read and approved the final manuscript.
